# Screening and diagnostic testing protocols for HIV and Syphilis infections in health care setting in Qatar: Evaluation and recommendations

**DOI:** 10.1371/journal.pone.0278079

**Published:** 2023-02-07

**Authors:** Gheyath K. Nasrallah, Raniya Al-Buainain, Nadin Younes, Soha R. Dargham, Duaa W. Al-Sadeq, Mohamed Elhassan, Ibrahim Al-Shaar, Hadi M. Yassine, Laith J. Abu-Raddad, Mohamed M. Emara, Ahmed Ismail

**Affiliations:** 1 Department of Biomedical Science, College of Health Sciences, QU Health, Qatar University, Doha, Qatar; 2 Biomedical Research Center, Qatar University, Doha, Qatar; 3 Medical Commission Department, Laboratory Section, Ministry of Public Health, Doha, Qatar; 4 Infectious Disease Epidemiology Group, Weill Cornell Medicine-Qatar, Cornell University, Doha, Qatar; 5 World Health Organization Collaborating Centre for Disease Epidemiology Analytics on HIV/AIDS, Sexually Transmitted Infections, and Viral Hepatitis, Weill Cornell Medicine–Qatar, Cornell University, Doha, Qatar; 6 College of Medicine, QU Health, Qatar University, Doha, Qatar; Children’s National Hospital, George Washington University, UNITED STATES

## Abstract

**Background:**

HIV and Syphilis are common STIs, which have become a concern and burden on healthcare systems, as many infections go untreated and lead to potentially serious complications. HIV is usually diagnosed with Western blot, PCR, and p24 antigen testing. Whereas, Syphilis is mainly diagnosed through clinical findings and serologic testing. The Medical Commission Department (MC) under MOPH is responsible for screening all newcomers to Qatar, aiming to keep the country free from serious infectious diseases.

**Objective:**

We aimed to evaluate the diagnostic efficiency of the protocols used in the MC for screening HIV and Syphilis infections.

**Methods:**

We conducted a retrospective study of samples analyzed by 4^th^ Generation ARCHITECT® HIV Ag/Ab Combo and Rapid Plasma Reagin (RPR) between January to December 2019. ARCHITECT® HIV Ag/Ab Combo positive samples were confirmed by INNO-LIA™ HIVI/II and RT-PCR. RPR-reactive samples were confirmed by ARCHITECT® Syphilis *Treponema pallidium* Antibody (Syphilis TPA) assay.

**Results:**

For HIV, data were collected from 585,587 individuals, of which 595 (0.1%) were positive by the ARCHITECT® HIV Ag/Ab Combo (Analyzer A). When all initially positive sera were re-tested on newly collected blood samples using different ARCHITECT® HIV Ag/Ab Combo analyzer (analyzer B), 99.8% (594/595) of samples were also positive, suggesting high reproducibility. The positive predictive value (PPV) between ARCHITECT® HIV Ag/Ab Combo and the INNO-LIA™ HIVI/II confirmatory assay was 31.8%. The PPV between ARCHITECT® HIV Ag/Ab Combo and HIV-PCR assay was 26.8%. Retrospective data for Syphilis were collected from a total of 97,298 individuals who visited the MC, of which 198 (0.20%) were initially positive by RPR. The PPV between RPR and Syphilis TPA confirmatory assay was 36.6%.

**Conclusion:**

Despite the high rate of false positivity using ARCHITECT® HIV Ag/Ab Combo and RPR screening assays, both assays have proven to be highly effective as screening testing methods.

## 1. Introduction

Sexually transmitted diseases (STDs) are a major public health concern and among the leading causes of morbidity and mortality worldwide, particularly in resource-poor settings [1]. The most common STDs include chlamydia, gonorrhea, trichomoniasis, acquired immunodeficiency syndrome (AIDS), and Syphilis, which are caused by *Chlamydia trachomatis*, *Neisseria gonorrhea*, *Trichomonas* vag*inalis*, Human immune deficiency virus (HIV), and *Treponema* palli*dum* (*T*. *pallidum*), respectively. In 2019, there were 1.7 million newly infected HIV individuals and 38 million [31.6 million–44.5 million] people living with HIV [2]. On the other hand, Syphilis, the oldest STD known to mankind, remains a major burden globally, impacting the quality of life, health and economies. Each year, there are an estimated 12 million new syphilis cases globally, with over 90% of the cases occurring in the developing world [3].

Given the fact that HIV and Syphilis are both STDs, co-infection is relatively common [4, 5]. Nearly 10% of Danish men were diagnosed with Syphilis acquired HIV infection within five years after their diagnosis with syphilis [6]. Thus, all patients diagnosed with Syphilis should be screened for HIV infection and vice versa [7]. In areas where HIV is highly prevalent, it is recommended that patients who were diagnosed with primary Syphilis get re-tested for HIV after 3 months if the first HIV test result was negative [8]. Epidemiological investigations have offered extensive evidence that syphilis infection facilitates HIV transmission [9–11]. For instant, syphilitic ulcers, which disrupt the epithelial and mucosal surfaces [9], can facilitate the entry of HIV virions [10]. Moreover, ulcerations can trigger an influx of CD4+ cells [10]. Furthermore, *T*. *pallidum* and its pro-inflammatory constituents can induce the expression of CCR5 (the major HIV coreceptor) on human monocytes in syphilitic lesions, thus increasing the susceptibility to HIV transmission [12]. The first six months following the estimated time of exposure to Syphilis represents the period of greatest risk for HIV infection [10]. Most importantly, Syphilis was found to be associated with increases in HIV viral loads and reduction in CD4+ cell counts [9].

Like most STDs, Syphilis and HIV are often asymptomatic; thus, sensitive diagnostic testing is necessary for early detection and diagnosis, serving as guidance for treatment. HIV can be diagnosed by detecting HIV antibodies [13] and/or detecting viral RNA, enzymes, and proteins [14] by applying different diagnostic technologies [8, 15]. Western Blot (WB) is considered the gold standard confirmatory test for the detection of specific HIV antibodies [16]. On the other hand, Syphilis can be diagnosed using a specific treponema dark field microscopy test [17]. However, its efficiency is limited [18]. Other test includes non-treponema test such as the RPR or serology tests such as *T*. *pallidum* hemagglutination assay (TPHA) are relatively insensitive in the early stage of infection. Neither non-treponemal tests nor treponemal tests can detect antibodies until the infection has progressed 1–3 weeks after the development of the chancre [18]. Unfortunately, qualitative nontreponemal tests are widely used for syphilis screening. However, their usefulness is limited by decreased sensitivity in early primary Syphilis and during late Syphilis, when up to one-third of untreated patients may be non-reactive [18]. Nevertheless, nontreponemal false-positive results are common because of pregnancy, autoimmune disorders, and infections [19]. Because of the drawback of each diagnostic technique for Syphilis, the CDC recommends using a nontreponemal antibody test for screening along with a treponemal antibody test for confirmation of syphilis infection [20].

According to the Qatar Ministry of Public Health (MOPH), communicable diseases account for around 8% of all deaths in Qatar, negatively affecting the life quality of residents and creating an overwhelming concern for Qatar’s healthcare system [21]. In addition, Qatar continues to face a considerable challenge in infection control. The Medical Commission (MC) in Qatar works under the umbrella of the MOPH, which contributes effectively to the implementation of the ministry’s general strategy. The MC is responsible for screening newcomers to Qatar to keep the country free from serious infectious diseases, including AIDS and Syphilis. To minimize the risk of importing and spreading serious infectious diseases, the MOPH requires individuals by law to take a medical exam upon arrival in the State.

In this study, we aimed to assess the protocol performances used for screening and diagnosing HIV and Syphilis in the MC laboratory in Qatar. Our work aimed to evaluate the current screening protocol and provide useful benchmark information to high authority if improvement of a screening protocol for HIV and Syphilis diagnosis is needed to achieve the most reliable results with minimal cost. The major advantage of our study is that samples were collected from individuals with different nationalities, hence, different prevalence of both diseases. In this study, we evaluated the screening and the diagnostic protocol for HIV by assessing the performance of ARCHITECT® HIV Ag/Ab Combo as a screening technology in comparison to the INNO-LIA™ HIV I/II (the gold standard test) and RT-PCR. Similarly, we evaluated the screening protocol for syphilis diagnosis by evaluating the performance of the rapid plasma reagin (RPR) test (screening test) and ARCHITECT® Syphilis TPA assay.

## 2. Methods

### 2.1. Ethical consideration

This study was approved by the Institutional review board (IRB) at Qatar University (QU-IRB 1242-E/20) and exemption under category 3 was obtained from the research department at Qatar Ministry of Public Health (MOPH).

### 2.2. Study population and eligibility criteria

This is a retrospective study. Thus, no patients/applicants were recruited and there was no direct or indirect interaction with any human subject. The study was conducted on existing de-identified testing results that were saved in the MC’s password-protected database medical system. Retrospective data was collected from a total of 585,587 individuals who were screened in the MC between January 1, 2019 and December 31, 2019.

### 2.3. HIV Ag/Ab combo test

The fully automated ARCHITECT® HIV Ag/Ab Combo (Abbott Diagnostics, Abbott Park, Illinois, U.S.A) is a qualitative antigen/antibody immunoassay. ARCHITECT® HIV Ag/Ab combo assay can simultaneously detect both the p24 antigen and antibody of HIV-1/HIV-2. However, this assay cannot distinguish between the detection of HIV p24 antigen, HIV-1 antibody, or HIV-2 antibody reactivity [22]. This assay was performed according to the manufacturer’s protocol. Briefly, human serum containing p24 antigens and antibodies were combined with a mixture of assay diluent, washing buffer, and paramagnetic microparticles coated with HIV-1/HIV-2 antigen and HIV p24 mouse monoclonal antibody. After that, the samples were washed with the washing buffer to get rid of any unbound complexes. Then, pre-trigger and trigger solutions were added to the reaction mixture and the resulting chemiluminescent reaction was measured as relative light units (RLUs). This signal indicated the presence or absence of the antigen or antibodies in the sample, and it was compared to the cut-off signal determined from the assay calibration. The signal to cut-off (S/CO) values determines if the sample is positive or negative for HIV. Samples showing S/CO values ≥ 1.00 were considered reactive (R) to HIV-1 p24 antigen or HIV-1/HIV-2 antibodies, whereas those ≤ 1.00 are non-reactive.

**Fig 1:**
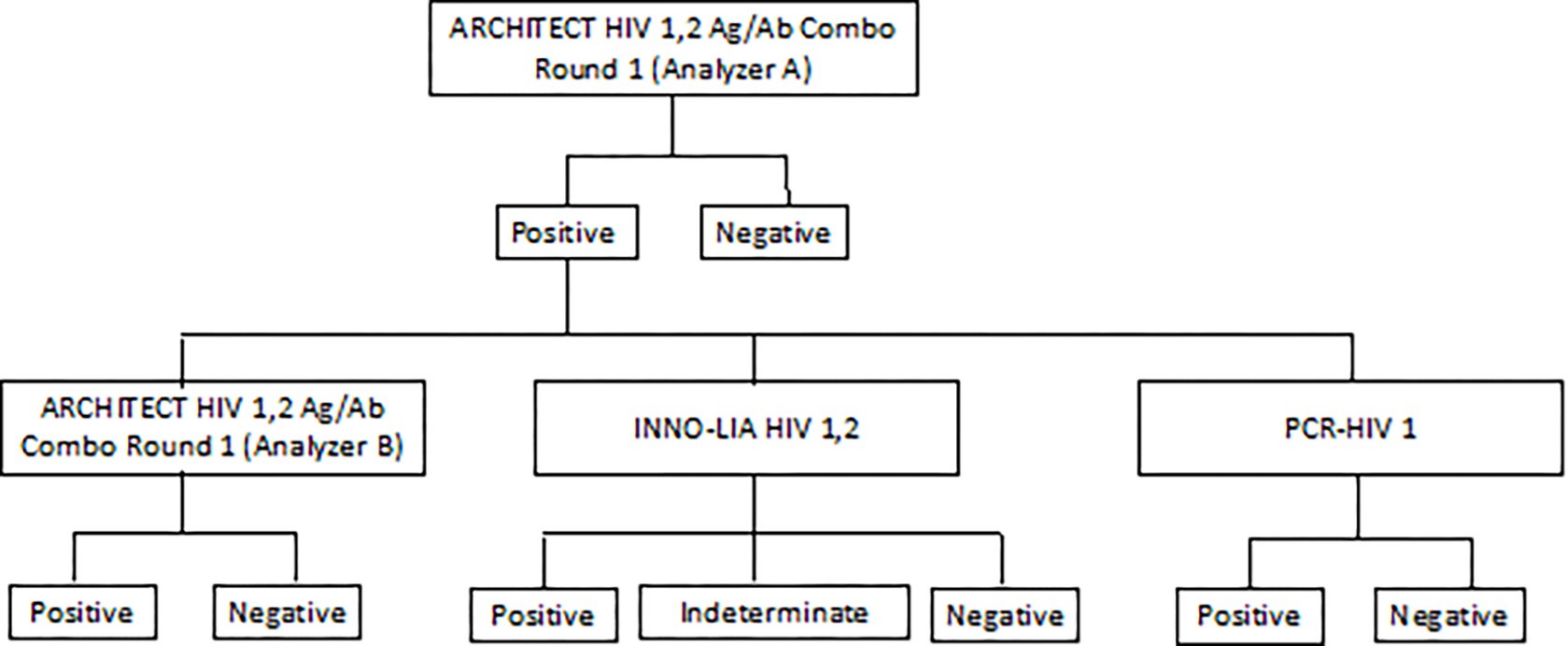
A chart summary of HIV diagnosis workflow. Analyzer B, INNO-LIA™ HIVI/II and PCR-HIV1 tests were done on a freshly drawn sample.

According to the MC Laboratory protocols, any sample that showed reactivity to HIV antigen and/or antibodies were tested again in duplicates. If both replicates were non-reactive, the result was considered non-reactive. If one or both replicates were reactive, the result should be considered reactive. All initial reactive samples from the HIV Ag/Ab Combo test (Run 1, analyzer A) were re-tested using another ARCHITECT® HIV Ag/Ab Combo (analyzer B) and 2 supplementary tests, namely INNO-LIA™ HIVI/II score and RT-PCR using freshly drawn blood samples (Run B) as shown in Fig 1.

### 2.4. INNO-LIA™ HIV I/II

To confirm the results of the ARCHITECT®HIV Ag/Ab combo assay (Run 1, Analyser A), all positive initial samples (Run 1) were re-tested using the gold standard INNO-LIA™ HIV I/II Score (Innogenetics, Ghent, Belgium) as a confirmatory test on a freshly drawn sample. This assay is a Line Immunoassay (LIA®) that could distinguish between HIV-1/HIV-2, where on a nylon strip, discrete lines of HIV-1 and HIV-2 recombinant proteins and synthetic peptides are coated. The structural proteins sgp120 and gp41, which detect specific HIV-1 antibodies, and p31, p24, and p17, which may also cross-react with HIV-2 antibodies. In addition, it also could detect Antibodies specific to HIV-2 gp36 and sgp105 [23]. The assay was performed using AUTO-LIA^TM^ 48 automated machine according to the manufacturer’s protocol. Briefly, 20 μL of the sample was added to 1ml of sample diluent (1:50) and incubated at room temperature (20°C) for 3 hours with the test strip, followed by three washing steps with washing buffer before the addition of a goat anti-human IgG conjugated to alkaline phosphatase. After incubation, three washing steps were again performed, followed by the addition of chromogen. Then, an appropriate stop solution was added to stop color development and the results were recorded. Afterwards, the result was interpreted using the LiRAS for infectious diseases software, which is designed to assist with the interpretation of the LIA results.

### 2.5. COBAS® AmpliPrep/COBAS® TaqMan® HIV-1 test, version 2.0

The COBAS® AmpliPrep/COBAS® TaqMan® HIV-1 Test (Roche Molecular Systems, Branchburg, USA) version 2.0 (v2.0) is an in-vitro nucleic acid amplification test for the quantitation of HIV-1 RNA in human plasma that targets two highly conserved regions of the HIV-1 genome, which are the gag and LTR regions. In doing so, it compensates for the possibility of mutations or mismatches and increases the probability of detection. This assay was performed according to the manufacturer’s protocol. The procedure processes 850μl of plasma sample. The COBAS® AmpliPrep/COBAS® TaqMan® HIV-1 Test, v2.0 is based on three major processes: (1) specimen preparation to separate HIV-1 RNA; (2) reverse transcription of the target RNA to produce complementary DNA (cDNA), and (3) simultaneous PCR amplification of target cDNA and the identification of cleaved dual-labeled oligonucleotide detection probe that is specific to the target. HIV-1 RNA was isolated with the COBAS-automated AmpliPrep extraction system. PCR amplification was then performed on the COBAS-automated TaqMan 48 system using the TaqMan v2.0 assay. The test can quantitate HIV-1 over the range of 20–10,000,000 copies/ml.

### 2.6. Rapid Plasma Reagin RPR test (Omega diagnostics of syphilis)

For the detection of Syphilis, we used the Rapid Plasma Reagin (RPR) card test (Omega Diagnostics Ltd, Scotland, UK). This is a qualitative and semi-quantitative non-treponemal flocculation assay used to detect reagin antibodies in the serum of patient samples. The principle of this assay is based on modified VDRL antigen that contains carbon particles for improving the results’ visualization. When binding occurs between reagin antibodies in the sample with cholesterol/ cardiolipin/ lecithin in the reagent and the reagin antibodies in the sample, the results can be seen macroscopically in the form of black clumps. No visual flocculation indicates a negative result.

**Fig 2:**
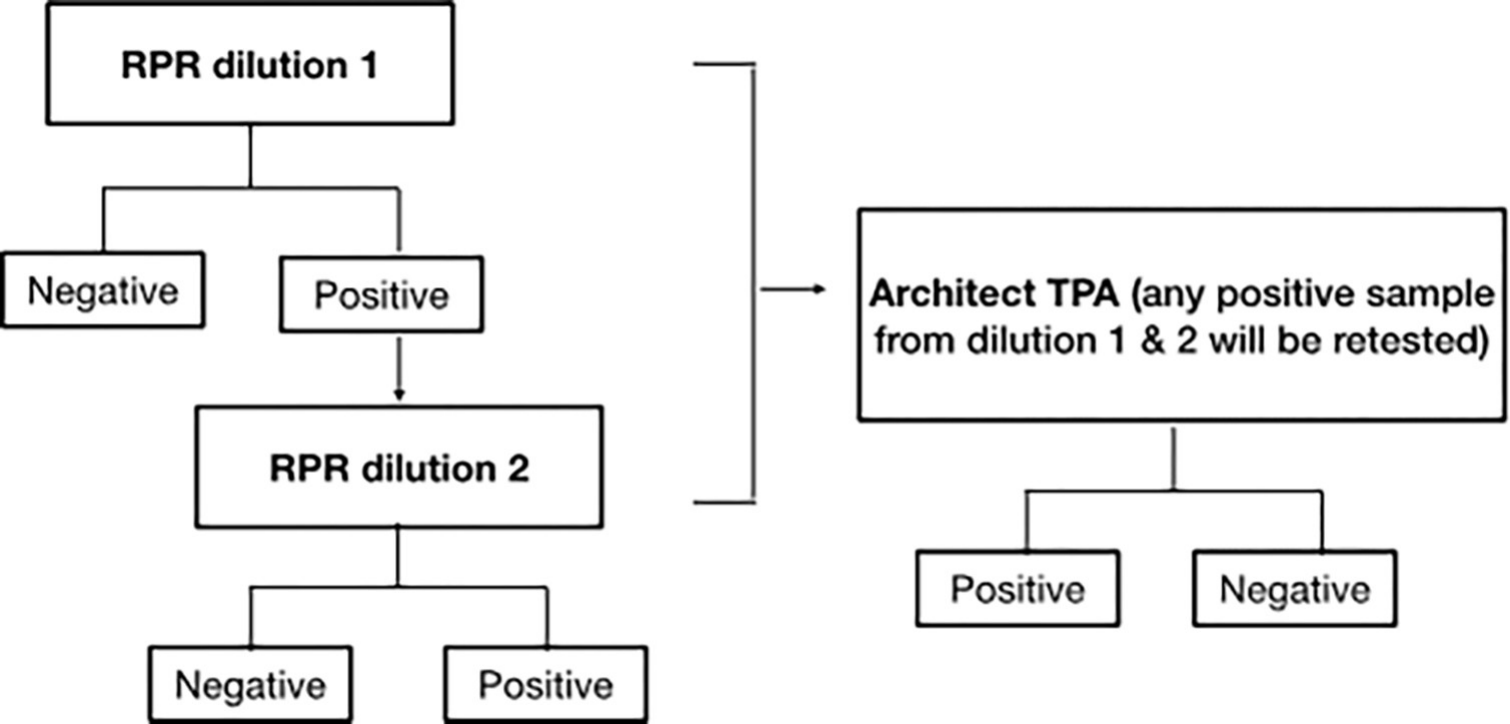
A chart summary of syphilis diagnosis workflow.

This assay was performed according to the manufacturer’s protocol. Briefly, 50 μL of the patient’s serum or plasma was dispensed and spread to cover a defined circle on the RPR test card. Then 16 μL of the antigen (provided by the manufacturer) was added to the sample and mixed by rotation at 100 rpm for 8 minutes on an automatic rotator. Black clumps formation was observed under the light. Positive samples were then subjected to semi-quantitative analysis. Briefly, serial dilutions (50 μL) of the patient’s serum were prepared using isotonic saline. Then, using the RPR test card, 16 μl of the antigen was added to one drop (16 μl) of diluted serum and mixed by rotation at 100 rpm for 8 minutes on an automatic rotator. The result is interpreted qualitatively as reactive if there are medium and large aggregates, weak reactive if there is finely dispersed aggregates, and non-reactive if there is no aggregates visible and only smooth grey appearance. The result was also interpreted semi-quantitatively as a titer of the last dilution produces a reactive result. According to the MC protocols, all reactive samples in RUN 1 will be re-checked by another technician for titer RUN 2. Any reactive samples from RPR run 1 and run 2 will be confirmed using ARCHITECT® Syphilis TP, as shown in Fig 2.

### 2.7. Statistical analysis

A descriptive statistical analysis of categorical variables was performed. The positive predictive values (PPV) was calculated to assess the performance of each assay. The significance level was indicated at 5%, and a 95% confidence interval (CI) was reported for each metric. All calculations were performed using Microsoft Excel 2016.

## 3. Results

### 3.1. The ARCHITECT® HIV Ag/Ab combo screening protocol showed excellent reproducibility

Retrospective data was collected from a total of 585,587 individuals who visited the MC between January 1, 2019 and December 31, 2019. Among the total of 585,587 individuals, we found 595 (0.1%) HIV-positive samples using the Architect HIV Ag/Ab Combo test (S1 Table). This data was obtained from two different runs using two different ARCHITECT® HIV Ag/Ab Combo (analyzers A and B) and two different blood samples drawn from the same individual on two different occasions (the 2-sample rule) in order to rule out any error in identification or sample mixing during blood drawing. The initial HIV screening using ARCHITECT® HIV Ag/Ab Combo (Run 1, Analyzer A) identified 595 positive HIV samples. For confirmation, all initial positive samples were re-tested again on freshly obtained blood samples using the HIV Ag/Ab test on a different ARCHITECT® HIV Ag/Ab Combo (Run 2, Analyzer B). From run 2, all positive samples except one (99.8%; 594/595) were also positive in run 2, suggesting excellent reproducibility of the Architect HIV Ag/Ab Combo Test as shown in Table 1.

**Table 1:** Comparison between ARCHITECT® HIV Ag/Ab combo run 1 and run 2.

ARCHITECT HIV Ag/Ab Combo Run 2 ARCHITECT HIV Ag/Ab Combo Run 1	Positive	Negative	Total
Positive	594	1	595
Negative*	N/A	N/A	N/A
Total	594	1	595

s*Only specimens that were reported positive in ARCHITECT HIV Ag/Ab Combo Run 1 were re-tested in ARCHITECT HIV Ag/Ab Combo Run 2

A total of 594 ARCHITECT® HIV Ag/Ab Combo positive samples were tested in INNO-LIA™ HIVI/II assay, while a total of 586 were tested in both confirmatory tests, INNO-LIA and HIV-1 PCR. Out of the 594 ARCHITECT® HIV Ag/Ab Combo positive samples, the INNO-LIA™ HIVI/II assay identified 173 (29.1%) as HIV-1 positive, 371 (62.5%) as HIV1,2 negatives, and 50 (8.41%) as indeterminate as shown in Table 2. Whereas the RT-PCR analysis identified 157 (26.8%) as HIV-1 positive and 429 (73.2%) negative ones, as shown in Table 3. 586 ARCHITECT® HIV Ag/Ab Combo positive samples were tested in both tests, namely INNO-LIA and HIV-1 PCR as shown in Table 4.

**Table 2:** Comparison between ARCHITECT® HIV Ag/Ab combo run 2 and INNO-LIA™ HIV I/II.

INNO-LIA™ HIV I/II ARCHITECT HIV Ag/Ab Combo Run 2	Positive	Negative	Indeterminate	Total
Positive	173	371	50	594
Negative*	N/A	N/A	N/A	N/A
Total	173	371	50	594

*Only specimens that were reported positive in ARCHITECT HIV Ag/Ab Combo Run 2 were re-tested with INNO-LIA™ HIV I/II

**Table 3:** Comparison between ARCHITECT® HIV Ag/Ab combo Run 2 and RT-PCR.

RT- PCR ARCHITECT HIV Ag/Ab Combo Run 2	Positive	Negative	Total
Positive	157	429	586
Negative*	N/A	N/A	N/A
Total	157	429	586

*Only specimens that were reported positive in ARCHITECT HIV Ag/Ab Combo Run 2 were re-tested with RT-PCR

**Table 4:** Comparison between INNO-LIA™ HIVI/II and PCR.

HIV1 RT-PCR INNO-LIA HIV I/II	Positive	Negative	Total
Positive	155	10	165
Negative	1	370	371
Indeterminate	1	49	50
Total	157	429	586

In 525 out of 586 samples (89.5%), there was an agreement between the two tests, and in 61 out of 586 samples (10.4%), there was a discrepancy. 155 out of 586 (26.5%) were positive by both tests and 370 out of 586 (63.1%) were negative by both tests. 49 out of 586 (8.36%) were indeterminate with INNO-LIA but negative with HIV-1 PCR. All those with indeterminate INNO-LIA were re-tested after 4 weeks (run 3), and the result remained the same. 10 out of 586 (1.7%) were positive for INNO-LIA HIV 1 but negative with HIV1-PCR. We do not have the treatment status of these 10 applicants who have HIV1-PCR negative; therefore, these results can be explained by either they controlled their viremia by treatment or they can be categorized as an elite controller that represents a rare group of individuals with an ability to maintain an undetectable HIV-1 viral load overtime in the absence of previous antiretroviral therapy. 1 out of 586 samples (0.2%) was positive for HIV1-PCR and indeterminate with INNO-LIA (could be in the seroconversion stage), and 1 out of 586 samples (0.2%) was positive with HIV1-PCR and negative with INNO-LIA test (in the window/ pre-seroconversion stage), which could be missed if 4^th^ generation HIV Ag/Ab was not used. All HIV-confirmed cases were having HIV-1 infection and no one reported having HIV-2 from the studied sample of 594

To ensure the results of ARCHITECT® HIV indicate the true positive cases, we calculated the positive predictive value (PPV). The PPV between ARCHITECT® HIV assay (Round 2, Analyzer B) & INNO-LIA™ HIV was 31.8% (95% CI; 28.3%-35.8%). Similarly, the PPV between ARCHITECT® HIV assay (Round 2, Analyzer B) and RT-PCR were low 26.8% (23.4%-30.5%). It is worth noting that PPV is affected by the prevalence of the disease. The screened population are coming from different countries with different prevalences of HIV infection. This might reflect the cause of the low PPV of ARCHITECT® HIV Ag/Ab Combo. The PPV could be improved by using Receiver Operating Characteristic Curve (ROC) analysis to determine the new cut-off value and reduce the unnecessary INNO-LIA and PCR tests (in this study, unfortunately, we do not have the data to calculate the ROC).

### 3.2. The RPR screening protocol shows high reproducibility with the confirmatory test

For syphilis analysis, data was obtained from the RPR test performed on 97,298 blood samples (collected between January 1, 2019 and December 31, 2019) to screen for the disease. Among the total of 97,298 individuals, we found 198 (0.20%) positive samples by RPR screening test (S2 Table). For validation RPR result, the test was done in two different runs (1 & 2) and by two different technicians without knowing the titer of each other. All 198 samples were reactive to Syphilis in both runs, as shown in Table 5, suggesting high reproducibility of the RPR assay (100%). All the RPR positive samples were re-tested using ARCHITECT® Syphilis TPA for confirmation. The TPA identified only 72 (36.6%, 72/198) positive samples for Syphilis and 125 negative samples, as shown in Table 6. The PPV between the RPR test and TPA-ELISA was 36.6% (95% CI; 30.1%-43.4%).

**Table 5:** Comparison between RPR run 1 and 2.

RPR run 2 RPR run 1	Positive	Negative	Total
Positive	198	0	198
Negative	N/A	N/A	N/A
Total	198	0	198

*Only specimens that were reported positive in RPR Run 1 were re-tested with RPR Run 2

**Table 6:** Comparison between RPR run 2 and TPA-ELISA.

ARCHITECT® Syphilis TPA RPR run 2	Positive	Negative	Total
Positive	72	125	197
Negative	N/A	N/A	N/A
Total	72	125	197

## 4. Discussion

One of the automated assays that are based on the CLIA principle and are widely used in many countries in the world, including Qatar is ARCHITECT® HIV Ag/Ab Combo immunoanalyzer [24]. ARCHITECT® HIV Ag/Ab Combo is an in vitro chemiluminescent microparticle (CMIA) that detects HIV p24 antigen and antibodies to HIV-1 and/or HIV-2 in human serum and plasma. It is fully automated and takes ~30-minutes time to detect the first result. According to the manufacturer, ARCHITECT® HIV Ag/Ab Combo overall specificity is 99.8% (95% CI: 99.6–99.9%), and HIV antibody sensitivity is 100% (95% CI: 99.6–100.0%). HIV Combo recognizes HIV diseases during the early, late, and intense stages of the infection [25]. It is used as an aid in the diagnosis of HIV-1/HIV-2 infection, including acute or primary HIV-1 infection. However, an ARCHITECT HIV Ag/Ab Combo reactive result does not distinguish between the detection of HIV-1 p24 antigen, HIV-1 antibody, or HIV-2 antibody. As with all immunoassays, the ARCHITECT HIV Ag/Ab Combo assay may yield nonspecific reactions due to other causes, particularly when testing in low prevalence populations.

In the present study, we were able to show that the Abbott ARCHITECT® HIV Ag/Ab Combo assay could be used efficiently in the MC for HIV screening. The assay performs with very high reproducibility (99.8%) with the two run repeats done on two different analyzers tested by two different technicians on two different samples taken on two different occasions from the same individuals. This reflects an excellent pre-analytical identification protocol that is being implemented by the pre-analytical unit. This high interpretation concordance indicates that the protocol used in the MC laboratory is highly precise in performing the assay as an initial screening for HIV detection and reflects an excellent pre-analytical identification protocol that is being implemented by the pre-analytical unit.

Our results are supported by the data obtained from different other ARCHITECT® HIV Ag/Ab Combo evaluations that were done on similar or higher numbers of samples from different countries [26–28]. A study conducted in Cameroon showed that the evaluated performance of the ARCHITECT® HIV Ag/Ab Combo kit (Abbott) was found to have 100% sensitivity and a specificity of 97.6% [24]. As with all immunoassays, it is worth mentioning that the ARCHITECT HIV Ag/Ab Combo assay could generate false positive results, particularly when testing in low prevalence populations. Therefore, A repeatedly reactive specimen should be investigated further with supplemental confirmatory HIV-specific tests, such as immunoblots, antigen tests, and HIV nucleic acid tests. On the other hand, testing an individual at the early stage of HIV infection may result in false-negative diagnoses [29–31]. Therefore, the optimal HIV testing method and algorithm are critical for screening the expatriates to avoid any missing cases of acute HIV infection from entering the country without detection. Moreover, the Medical commission laboratory is screening around 2500 applicants per day. In order to avoid any error in sampling, it follows the protocol of re-testing any initial reactive samples, on freshly drawn blood samples using another ARCHITECT® HIV Ag/Ab Combo analyzer and at the same time confirm it by INNO-LIA and RT-PCR. This is extremely important because it is significantly decrease the chances of a false positive diagnosis, which in-turn decreases the number of people who can potentially receive wrong information about their health status and reduce the psychological impact of misdiagnosis and its implications on the individuals, their partners and social contacts.

In 2014 the CDC and the Association of Public Health Laboratories published a new laboratory algorithm for the diagnosis of HIV [32]. The algorithm starts with 4^th^ generation HIV test, followed by HIV-1/HIV-2 antibody differentiation Immunoassay, followed by Nucleic Acid Testing (NAT). The medical commission laboratory using the same algorithm starting with 4^th^ generation ARCHITECT® HIV Ag/Ab Combo followed by INNO-LIA HIV I/II score as antibody differentiation immunoassay and NAT. The primary advantage of the new algorithm is the ability to identify HIV infection earlier [33, 34]. This is critical because the risk of HIV transmission from persons with acute and early infection is much higher than that from person with established infections. Therefore, identifying these cases as early as possible and initiating antiretroviral therapy (ART) can benefit patients and reduce HIV transmission.

The comparison between ARCHITECT® HIV Ag/Ab Combo results and the INNO-LIA HIV I/II showed a low PPV (29.1%), and the majority 62.5% were negative in INNO-LIA, which is the gold standard test. In addition, all INNO-LIA HIVI/II HIV1,2 negative and indeterminate samples were also negative with RT-PCR except two which could be either in the window or seroconversion period. The high number of false positive ARCHITECT® HIV Ag/Ab Combo could be explained by the fact that our screened population coming from various parts of the world with different prevalence. 8.4% of ARCHITECT® HIV Ag/Ab Combo positive samples got an indeterminate result which remained the same after 4 weeks of re-testing on in INNO-LIA. Almost all INNO-LIA positive samples were due to HIV 1. This could be explained by the fact that there are few newcomers to Qatar from west Africa, where HIV 2 is prevalent. The CDC is using the FDA-approved Bio-Rad Geenius HIV1,2 assay as antibody differentiation. In the MC lab, INNO-LIA CE approved assay is used as antibody differentiation test with around 8% indeterminate result. Orna et al found that Geenius assay reduced the number of indeterminate results in comparison to INNO-LIA which could be evaluated in our lab [35, 36].

29.1% of ARCHITECT® HIV Ag/Ab Combo positive samples were positive using INNO-LIA HIV I/II antibody and 26.8% were positive using RT-PCR. Almost all samples which are positive with INNO-LIA HIV I/II, are positive with HIV-1 RT-PCR except 10 samples (1.7%), which could be explained by either they controlled their viremia by treatment or they can be categorized as elite controller that represents a rare group of individuals with an ability to maintain an undetectable HIV-1 viral load overtime in the absence of previous antiretroviral therapy. The probability of having HIV 2 infection has been ruled out by INNO-LIA HIV I/II. Almost all HIV-1 RT-PCR positive samples are positive using INNO-LIA H I/II except 2 cases which indicate that they are in the window or seroconversion period, a result that illustrates the importance of using 4^th^ generation HIV Ag/Ab test in MC protocol which leads to decreasing the window period and improving early detection of HIV infection. Our study showed a high level of false positive ARCHITECT® HIV Ag/Ab Combo results that indicated by the fact that around 63% of positive samples were negative in both INNO-LIA and RT-PCR. This result necessitates the lab decision-maker to revise the cut-off value decided by the manufacturer without affecting the sensitivity or specificity of the test [37–40].

Given that Qatar’s MC is the main hub in the country to screen individuals coming from different parts of the globe makes it is essential to utilize tools that can detect all subtypes of HIV, have fast turn-around time and are cost-efficient. Thus, the high throughput of ARCHITECT® HIV Ag/Ab Combo analyzer remains the preferable technique used for massive screening of HIV as it is not cost-effective to apply neither RT-PCR nor line Immunoassay as the primary tool for massive screening for HIV.

Syphilis is an important infection that causes serious health problems mainly sexually transmitted disease, neurosyphilis and congenital infection. Therefore, appropriate screening, confirmation and follow-up protocols are required. MC laboratory follows the traditional Syphilis screening algorithm, which starts with a non-treponemal reagin test (RPR) as the first-line diagnostic approach, followed by a treponemal test (TPA) as a confirmatory test [41, 42]. Start with the RPR test as the first-line, which is cost-effective and reliable in various prevalence settings. Reactivity of a non-treponemal test such as RPR indicates the activity of the disease, which is very important from a public health perspective, while reactivity of a treponemal test cannot differentiate between current and past infection. Moreover, RPR can be used to follow treatment efficacy and assess disease recurrence. However, the RPR test has some pitfalls, including manual test, and the result interpretation is subjective depending on the operator. There have been efforts to automate the RPR testing. RPR tests may show false positive results for various reasons, including lupus, viral mononucleosis, malaria, leprosy, viral pneumonia, and rickettsia infection. In order to reduce the effect of RPR interpretation subjectivity, the MC implemented a protocol of repeating all initial reactive RPR to be done by another operator and compare the result before going to the next step of more expensive treponemal (TPA) test. When comparing the two runs for all reactive RPR done by two different technicians, the results showed that there is 100% agreement between the two runs in reactivity and titer (dilution), as shown in Table 6. The PPV of the RPR test is only 36.5%, with more than 50% of RPR reactive samples being negative in TPA confirmatory test. This high false positive result could be explained by the fact that the screened population came from various parts of the world with different disease prevalences.

## 5. Conclusion

The screening protocols for HIV and Syphilis used in the MC are highly reliable, reproducible and efficient for the screening of STDs. The Architect HIV Ag/Ab Combo Assay, a fourth-generation ELISA, has proven to be highly reliable for screening HIV infection. However, its high sensitivity may lead to false-positive results. Thus, according to the MC protocol, using confirmatory tests such as INNO-LIA HIV I/II and RT-PCR are critical as it significantly decreases the chances of false positives, which in turn reduces the number of people who can potentially receive wrong information about their health status. MC Lab uses the traditional testing algorithm for syphilis screening, which begins with a non-treponemal conventional manual RPR card test followed by a confirmatory treponemal test ARCHITECT® Syphilis TP for reactive RPR results. This algorithm costs less than the reverse algorithm, which begins with a treponemal test. Furthermore, non-treponemal test reactivity, such as RPR, indicates disease activity, which is critical from a public health standpoint, whereas treponemal test reactivity cannot distinguish between current and past infection. Our study found that the RPR test is a reliable and cost-effective method. However, due to the large number of samples (250 to 300 per day), the MC lab is currently working on automating the RPR test. RPR has some limitations, such as a prozone reaction may occur on rare occasions. Also, biological false positive RPR can occur in non-syphilitic conditions such as malaria, leprosy, rheumatoid arthritis etc. Thus, using a confirmatory test such as ARCHITECT® Syphilis TPA is critical, which is done according to the MC protocol.

## 6. Limitation

One of the limitations of this study is that we do not have data about HIV and Syphilis co-infection among the screened study population. One of the reasons is that not all applicants screened for HIV will be screened for Syphilis at the same time. But all applicants screened for Syphilis were screened for HIV, but we do not have the HIV status in this study as we did not think of it at the beginning of the study and our concentration on the screening protocol.

## Supporting information

S1 Table Screening results of INNO-LIA, ARCHITECT and rt-PCR for the detection of HIV. (XLSX)

S2 Table Screening results of RPR and TPA assays for the detection of Syphilis. (XLSX)

## References

[1] (WHO) WHO. Guidelines for the management of sexually transmitted infections 2003

[2] UNAIDS. Global HIV & AIDS statistics—2020 fact sheet 2019. Available from: https://www.unaids.org/en/resources/fact-sheet.

[3] Hook EW, 3rd, Peeling RW. Syphilis control—a continuing challenge. N Engl J Med. 2004;351(2):122–4. 15247352 10.1056/NEJMp048126

[4] Kojima N, Klausner JD. An Update on the Global Epidemiology of Syphilis. Current epidemiology reports. 2018;5(1):24–38. doi: 10.1007/s40471-018-0138-z 30116697 PMC6089383

[5] Mandel G BJ, Dolin R. Treponema pallidum (Syphilis). Principles and practice of Infectious Disease. Sixth Edition ed2005.

[6] Salado-Rasmussen K. Syphilis and HIV co-infection. Epidemiology, treatment and molecular typing of Treponema pallidum. Danish medical journal. 2015;62(12):B5176. 26621404

[7] Karumudi UR, Augenbraun M. Syphilis and HIV: a dangerous duo. Expert Rev Anti Infect Ther. 2005;3(5):825–31. doi: 10.1586/14787210.3.5.825 16207174

[8] CDC. Syphilis 2015. Available from: https://www.cdc.gov/std/tg2015/syphilis.htm.

[9] Buchacz K, Patel P, Taylor M, Kerndt PR, Byers RH, Holmberg SD, et al. Syphilis increases HIV viral load and decreases CD4 cell counts in HIV-infected patients with new syphilis infections. Aids. 2004;18(15):2075–9. doi: 10.1097/00002030-200410210-00012 15577629 PMC6763620

[10] Reynolds SJ, Risbud AR, Shepherd ME, Rompalo AM, Ghate MV, Godbole SV, et al. High rates of Syphilis among STI patients are contributing to the spread of HIV-1 in India. Sex Transm Infect. 2006;82(2):121–6. doi: 10.1136/sti.2005.015040 16581736 PMC2564682

[11] Fleming DT, Wasserheit JN. From epidemiological synergy to public health policy and practice: the contribution of other sexually transmitted diseases to sexual transmission of HIV infection. Sex Transm Infect. 1999;75(1):3–17. doi: 10.1136/sti.75.1.3 10448335 PMC1758168

[12] Sellati TJ, Wilkinson DA, Sheffield JS, Koup RA, Radolf JD, Norgard MV. Virulent Treponema pallidum, lipoprotein, and synthetic lipopeptides induce CCR5 on human monocytes and enhance their susceptibility to infection by human immunodeficiency virus type 1. J Infect Dis. 2000;181(1):283–93. doi: 10.1086/315209 10608777

[13] Bhowan K, Kalk E, Khan S, Sherman G. Identifying HIV infection in South African women: How does a fourth generation HIV rapid test perform? African journal of laboratory medicine. 2012;1(1):4. doi: 10.4102/ajlm.v1i1.4 29062724 PMC5644524

[14] Fearon M. The laboratory diagnosis of HIV infections. Can J Infect Dis Med Microbiol. 2005;16(1):26–30. doi: 10.1155/2005/515063 18159524 PMC2095005

[15] Jentsch U, Lunga P, Lacey C, Weber J, Cairns J, Pinheiro G, et al. The implementation and appraisal of a novel confirmatory HIV-1 testing algorithm in the Microbicides Development Programme 301 Trial (MDP301). PloS one. 2012;7(9):e42322. doi: 10.1371/journal.pone.0042322 22984401 PMC3439440

[16] Huang X, Liu X, Chen J, Bao Y, Hou J, Lu X, et al. Evaluation of Blood-Based Antibody Rapid Testing for HIV Early Therapy: A Meta-Analysis of the Evidence. Front Immunol. 2018;9:1458–. doi: 10.3389/fimmu.2018.01458 30013552 PMC6036269

[17] Larsen SA, Steiner BM, Rudolph AH. Laboratory diagnosis and interpretation of tests for Syphilis. Clin Microbiol Rev. 1995;8(1):1–21. doi: 10.1128/CMR.8.1.1 7704889 PMC172846

[18] Cummings MC, Lukehart SA, Marra C, Smith BL, Shaffer J, Demeo LR, et al. Comparison of methods for the detection of treponema pallidum in lesions of early syphilis. Sex Transm Dis. 1996;23(5):366–9. doi: 10.1097/00007435-199609000-00004 8885066

[19] Tsang RSW, Martin IE, Lau A, Sawatzky P. Serological diagnosis of Syphilis: comparison of the Trep-Chek IgG enzyme immunoassay with other screening and confirmatory tests. FEMS Immunology & Medical Microbiology. 2007;51(1):118–24. doi: 10.1111/j.1574-695X.2007.00289.x 17854473

[20] CDC. Sexually Transmitted Disease Treatment Guidlines 2015

[21] MOPH. Communicable diseases. Available from: https://www.moph.gov.qa/english/strategies/Supporting-Strategies-and-Frameworks/QatarPublicHealthStrategy/Pages/Communicable-diseases.aspx.

[22] Diagnostics A. ARCHITECT HIV Ag/Ab Combo Reagent Insert.

[23] WHO. WHO Prequalification of In Vitro Diagnostics Programme PUBLIC REPORT: NNO-LIA HIV I/II Score. 2015.

[24] Kfutwah A, Lemée V, Ngono HV, De Oliveira F, Njouom R, Plantier JC. Field evaluation of the Abbott ARCHITECT HIV Ag/Ab Combo immunoassay. Journal of clinical virology: the official publication of the Pan American Society for Clinical Virology. 2013;58 Suppl 1:e70–5. doi: 10.1016/j.jcv.2013.08.015 24342480

[25] Branson C B. HIV Diagnostics: New Tests and New Algorithms 2011. Available from: https://www.theaidsinstitute.org/sites/default/files/attachments/Brennan.pdf.

[26] Patel P, Mackellar D, Simmons P, Uniyal A, Gallagher K, Bennett B, et al. Detecting acute human immunodeficiency virus infection using 3 different screening immunoassays and nucleic acid amplification testing for human immunodeficiency virus RNA, 2006–2008. Archives of internal medicine. 2010;170(1):66–74. doi: 10.1001/archinternmed.2009.445 20065201

[27] Pandori MW, Hackett J, Louie B, Vallari A, Dowling T, Liska S, et al. Assessment of the ability of a fourth-generation immunoassay for human immunodeficiency virus (HIV) antibody and p24 antigen to detect both acute and recent HIV infections in a high-risk setting. Journal of clinical microbiology. 2009;47(8):2639–42. doi: 10.1128/JCM.00119-09 19535523 PMC2725691

[28] Stekler JD, Swenson PD, Coombs RW, Dragavon J, Thomas KK, Brennan CA, et al. HIV testing in a high-incidence population: is antibody testing alone good enough? Clinical infectious diseases. 2009;49(3):444–53. doi: 10.1086/600043 19538088 PMC3361648

[29] Chavez P, Wesolowski L, Patel P, Delaney K, Owen SM. Evaluation of the performance of the Abbott ARCHITECT HIV Ag/Ab Combo Assay. Journal of clinical virology: the official publication of the Pan American Society for Clinical Virology. 2011;52 Suppl 1:S51–5. doi: 10.1016/j.jcv.2011.09.010 21983253

[30] Mühlbacher A, Schennach H, Van Helden J, Hebell T, Pantaleo G, Bürgisser P, et al. Performance evaluation of a new fourth-generation HIV combination antigen–antibody assay. Medical microbiology and immunology. 2013;202(1):77–86. doi: 10.1007/s00430-012-0250-5 22706797 PMC3562432

[31] Alonso R, Pérez-García F, Gijón P, Collazos A, Bouza E. Evaluation of the Architect HIV Ag/Ab Combo Assay in a low-prevalence setting: The role of samples with a low S/CO ratio. Journal of clinical virology: the official publication of the Pan American Society for Clinical Virology. 2018;103:43–7. doi: 10.1016/j.jcv.2018.04.002 29635210

[32] Centers for Disease Control and Prevention and Association of Public Health Laboratorie. Laboratory Testing for the Diagnosis of HIV Infection: Updated Recommendations 2014. Available from: http://stacks.cdc.gov/view/cdc/23447.

[33] Laboratories CfDCaPaAoPH. Laboratory Testing for the Diagnosis of HIV Infection: Updated Recommendations 2014. Available from: http://stacks.cdc.gov/view/cdc/23447.

[34] Delaney KP, Heffelfinger JD, Wesolowski LG, Owen SM, Meyer WA 3rd, Kennedy S, et al. Performance of an alternative laboratory-based algorithm for HIV diagnosis in a high-risk population. Journal of clinical virology: the official publication of the Pan American Society for Clinical Virology. 2011;52 Suppl 1:S5–10. doi: 10.1016/j.jcv.2011.09.013 22019251

[35] Meles H, Wolday D, Fontanet A, Tsegaye A, Tilahun T, Aklilu M, et al. Indeterminate human immunodeficiency virus Western blot profiles in ethiopians with discordant screening-assay results. Clin Diagn Lab Immunol. 2002;9(1):160–3. doi: 10.1128/cdli.9.1.160-163.2002 11777847 PMC119890

[36] Mor O, Mileguir F, Michaeli M, Levy I, Mendelson E. Evaluation of the Bio-Rad Geenius HIV 1/2 assay as an alternative to the INNO-LIA HIV 1/2 assay for confirmation of HIV infection. J Clin Microbiol. 2014;52(7):2677–9. doi: 10.1128/JCM.01184-14 24789189 PMC4097735

[37] Albertoni G, Girao MJBC, Schor N. Mini review: current molecular methods for the detection and quantification of hepatitis B virus, hepatitis C virus, and human immunodeficiency virus type 1. International Journal of Infectious Diseases. 2014;25:145–9. 24927665 10.1016/j.ijid.2014.04.007

[38] Al Soub H, Al-khal AL, Al Maslamani M, Dousa K, Ahmed A, Fabella A. Epidemiology and the changing face of HIV infection in Qatar. Infectious Diseases in Clinical Practice. 2018;26(4):220–3.

[39] Sharp PM, Hahn BH. Origins of HIV and the AIDS pandemic. Cold Spring Harbor perspectives in medicine. 2011;1(1):a006841. doi: 10.1101/cshperspect.a006841 22229120 PMC3234451

[40] Rodrigo C, Rajapakse S. Current status of HIV/AIDS in South Asia. Journal of global infectious diseases. 2009;1(2):93. doi: 10.4103/0974-777X.56249 20300398 PMC2840955

[41] Dunseth CD, Ford BA, Krasowski MD. Traditional versus reverse syphilis algorithms: A comparison at a large academic medical center. Pract Lab Med. 2017;8:52–9. doi: 10.1016/j.plabm.2017.04.007 28856228 PMC5575410

[42] Lee J-H, Lim CS, Lee M-G, Kim H-S. Comparison of an automated rapid plasma reagin (RPR) test with the conventional RPR card test in syphilis testing. BMJ Open. 2014;4(12):e005664. doi: 10.1136/bmjopen-2014-005664 25552608 PMC4281540

